# The Frequency of Design Studies Targeting People With Psychotic Symptoms and Features in Mental Health Care Innovation: Secondary Analysis of a Systematic Review

**DOI:** 10.2196/54202

**Published:** 2024-01-09

**Authors:** Lars Veldmeijer, Gijs Terlouw, Jim Van Os, Job Van 't Veer, Nynke Boonstra

**Affiliations:** 1 Department of Psychiatry Utrecht University Medical Center Utrecht Netherlands; 2 Department of Healthcare and Welfare NHL Stenden University of Applied Sciences Leeuwarden Netherlands; 3 KieN VIP Mental Health Care Services Leeuwarden Netherlands

**Keywords:** design approaches, design, innovation, innovative, innovate, innovations, psychiatry, mental health care, mental health, mental illness, mental disease, involvement, service users, people with lived experience, people with lived experiences, lived experience, lived experiences, co-creation, cocreation, psychosis, psychotic, schizophrenia, schizoid, schizotypal, paranoia, neurosis, hallucinosis, hallucination, hallucinations

## Abstract

This study examined and reflected on the frequency of people with psychotic symptoms and features as the target population in design studies for mental health care innovation.

## Introduction

There is growing evidence highlighting the importance of involving people with lived experience in design processes in mental health care [1,2]. Particular attention should be directed toward the engagement of people with psychotic symptoms and features [3], as they often feel misunderstood due to their altered perceptions and subjective experiences [4,5]. A bottom-up review of the lived experience of psychosis emphasizes the complexity of psychotic symptoms and features and recommends including lived experience in designing mental health services to address these experiences and needs [6]. Design approaches can promote the involvement of people with firsthand experiences in the development of treatment, therapy, and recovery interventions for mental health care innovation [2]. Currently, it is unknown how frequent design studies specifically target people with psychotic symptoms and features. There is a scoping review of coproducing research on psychosis [7], but coproduction and design approaches are distinct methodologies. Design approaches facilitate designing initiatives that prioritize participants’ needs, expertise, and knowledge whereas coproduction facilitates collaborative delivery and knowledge production. In this research letter, we present findings on the frequency of design studies targeting people with psychotic symptoms by analyzing a prior systematic review data set that focused on involving people with firsthand experiences in designing mental health care innovations. The primary objective of this secondary data analysis was to elucidate how often design studies in mental health care target people with psychotic symptoms and features.

## Methods

### Primary Data Set and Secondary Data Analysis

We conducted a secondary data analysis using a data set from a prior systematic review that assessed the involvement of service users and people with lived experience in the design processes of mental health care innovation. In the screening process and study selection of the prior systematic review, 33 papers met the inclusion criteria [2]. All included papers were original reports or papers that (1) involved service users, people with lived experience, or both; (2) mentioned design approaches; (3) involved an empirical study; and (4) conducted the study in settings including mental health care services or psychiatry programs. In this secondary analysis, we examined the primary data set to provide an overview of the frequency of design studies in mental health care focusing on people with psychotic symptoms and features. This data set is suitable for this analysis since the search strategy of the systematic review did not target specific mental health conditions.

### Data Extraction and Categorization

Studies were categorized based on their primary target population as reported in the studies (Multimedia Appendix 1). We categorized broad terms like psychosis, which encompasses various symptoms and features like altered perceptions, as well as mental health conditions in which psychotic symptoms and features are prevalent, such as schizophrenia. In the *Diagnostic and Statistical Manual of Mental Disorders, Fifth Edition, Text Revision* (DSM-5-TR), these symptoms, features, and conditions fall under the category “schizophrenia spectrum and other psychotic disorders,” covering a spectrum of related mental health conditions [8], also referred to as the psychosis spectrum [9]. Studies addressing psychotic symptoms and features or related conditions alongside unrelated mental health conditions were labeled “various mental health conditions” due to their comorbid nature. We did not count these studies as primarily focusing on people with psychotic symptoms and features. Studies not centered on psychotic symptoms or related conditions were also categorized to determine the frequency of studies that target people with psychotic symptoms and features in the data set.

### Research Question

We aimed to answer the following research question: how frequently are people with psychotic symptoms and features the target group in design studies that involve service users or people with lived experience in mental health care innovation?

## Results

### The Frequency of Design Studies Targeting People With Psychotic Symptoms and Features

Of the studies in the data set that focused on specific target populations, 18% (6/33) centered on psychosis and 9% (3/33) concentrated on schizophrenia. Since psychosis and schizophrenia are considered part of a broader defined spectrum of psychotic-related mental health conditions in which psychotic symptoms and features are prevalent, the total proportion of studies that primarily focused on people with psychotic symptoms and features was 27% (9/33). The largest group of studies, accounting for 34% (11/33) of the data set, did not focus on specific target populations and were classified as “various mental health conditions.” In this category, 12% (4/33) included psychotic symptoms and features as a target in addition to other mental health conditions (Table 1).

**Table 1:** Target populations in mental health design studies.

Target population	Count, n (%)
Psychosis	6 (18)
Depression	4 (12)
Schizophrenia	3 (9)
Self-harm	2 (6)
Eating disorders	2 (6)
Substance use disorders	1 (3)
Borderline	1 (3)
Attention-deficit/hyperactivity disorder	1 (3)
Autism spectrum disorder	1 (3)
Bipolar disorder	1 (3)
Various mental health conditions	11 (34)

## Discussion

This secondary data analysis revealed a notable emphasis on studies primarily targeting people with psychotic symptoms and features in mental health care design studies. This is noteworthy given the extensive range of mental health conditions in psychiatry, encompassing 21 categories according to the DSM-5-TR [8]. Although “schizophrenia spectrum and other psychotic disorders” constitutes only 4.67% (1/21) of these categories, 27% (9/33) of studies in our data set focused primarily on psychotic symptoms and features. This percentage is high, considering the lifetime prevalence of psychotic disorders is approximately 1% [10]. Another 12% (4/33) of the studies mention psychotic symptoms and features alongside or as a result of other mental health conditions. Although these studies did not focus primarily on people with psychotic symptoms and features, they have shown that much attention has been given to psychotic experiences in design studies. The substantial research focus on people with psychotic symptoms and features in design studies may be attributed to the limited progress in prognosis for severe cases despite extensive research and treatment efforts [11]. This may prompt designers and researchers to look for less conventional strategies to enforce novel promising solutions. Additionally, there is a growing call for attention to the subjective experience of psychotic symptoms and features in clinical care, as these vary from individual to individual (eg, [4-6]). Both factors underscore the urgency of involving people with firsthand experiences to capture the vividness of psychotic experiences in the design of innovative services and interventions, ultimately aiming to improve outcomes for service users.

Comparing the 9 studies that primarily focused on people with psychotic symptoms and features in this secondary data analysis to the results of the prior systematic review, we observed that 44% (4/9) demonstrated a high level of participant involvement in their design processes [2]. This is crucial for the development of new innovations because research shows psychotic symptoms and features can seem very different from a lived experience perspective compared to conventional psychiatric conceptualizations [6]. At the same time, the results stress the ongoing need to engage people with lived experience of psychotic symptoms and features in design studies, as more than half of the studies did not show the substantial involvement that would be expected of design processes that aim to tailor innovations to the needs of the target group. Consequently, we recommend future design studies targeting people with psychotic symptoms and features to adopt the co-design methodology, as co-design shows the highest participant involvement levels in mental health care design studies [2]. Furthermore, researchers are encouraged to use the participation matrix [12] alongside co-design to make intentional methodological decisions regarding the phases and roles in which people with lived experience are involved. To prevent tokenism and cooptation in design processes, researchers and designers are recommended to systematically coreflect with people with lived experience, exploring the roles played and distilling benefits and challenges from both perspectives.

## Appendix

Multimedia Appendix 1 Categorization of studies based on their target population.

## Abbreviations

DSM-5-TR: *Diagnostic and Statistical Manual of Mental Disorders, Fifth Edition, Text Revision*
