# Supplementary material for: The Frequency of Design Studies Targeting People With Psychotic Symptoms and Features in Mental Health Care Innovation: Secondary Analysis of a Systematic Review
Source: JMIR Ment Health. 2024 Jan 9;11:e54202. doi: 10.2196/54202 (PMC10807378; doi:10.2196/54202)
Supplement: Multimedia Appendix 1 [file mental_v11i1e54202_app1.docx]

Appendix 1. Design studies, target populations and publication dates

| Author(s) | Title | Target population(s) | Publication date |
| --- | --- | --- | --- |
| Nakarada-Kordic, Hayes, Reay, Corbet and Chan | Co-designing for mental health: creative methods to engage young people experiencing psychosis | Psychosis | 2017 |
| McCLelland and Fitzgerald | A participatory mobile application (app) development project with mental health service users and clinicians | Psychosis | 2018 |
| Romm, Gardsjord, Gjermundsen, Ulloa, Berentzen and Melle | Designing easy access to care for first-episode psychosis in complex organizations. | Psychosis | 2019 |
| Realpe, Elahi, Bucci, Birchwood, Vlaev, Taylor and Thompson | Co-designing a virtual world with young people to deliver social cognition therapy in early psychosis. | Psychosis | 2019 |
| Knight, West, Matthews, Kabir, Lambe, Waite and Freeman | Participatory design to create a VR therapy for psychosis | Psychosis | 2021 |
| Illarregi, Alexiou, DiMalta and Zamenopoulos | Is designing therapeutic? A case study exploring the experience of co-design and psychosis | Psychosis | 2022 |
| Fonseka, Pong, Kcomt, Kennedy and Parikh | Collaborating with individuals with lived experience to adapt CANMAT clinical depression guidelines into a patient treatment guide: The CHOICE-D co-design process. | Depression | 2019 |
| Stawarz, Preist, Tallon, Wiles, Kessler, Turner, Shafran and Coyle | Design considerations for the integrated delivery of cognitive behavioral therapy for depression: User-centered design study | Depression | 2020 |
| Callan, Dunbar Jacob, Siegle, Dey, Thase, DeVito Dabbs, Kazantzis, Rotondi, Tamres, van Slyke and Sereika | CBT MobileWork©: User-centered development and testing of a mobile mental health application for depression | Depression | 2020 |
| Jenness, Bhattacharya, Kientz, Munson and Nagar | Lessons learned from designing an asynchronous remote community approach for behavioral activation intervention for teens | Depression | 2022 |
| Terp, Laursen, Jørgensen, Mainz and Bjørnes | A room for design: Through participatory design young adults with schizophrenia become strong collaborators. | Schizophrenia | 2016 |
| Terp, Jørgensen, Laursen, Mainz and Bjørnes | A smartphone app to foster power in the everyday management of living with schizophrenia: Qualitative analysis of young adults' perspectives | Schizophrenia | 2018 |
| García, Fernández-Sotos, Vicente-Querol, Sánchez-Reolid, Rodriguez-Jimenez and Fernández-Caballero | Co-design of avatars to embody auditory hallucinations of patients with schizophrenia: A study on patients’ feeling of satisfaction and psychiatrists’ intention to adopt the technology | Schizophrenia | 2021 |
| Owens, Farrand, Darvill, Emmens, Hewis and Aitken | Involving service users in intervention design: A participatory approach to developing a text‐messaging intervention to reduce repetition of self‐harm | Self-harm | 2010 |
| Kruzan, Meyerhoff, Biernesser, Goldstein, Reddy, Mohr | Centering Lived Experience in Developing Digital Interventions for Suicide and Self-injurious Behaviors: User-Centered Design Approach. | Self-harm | 2022 |
| Milton, Hambleton, Downling, Roberts, Davenport and Hickie | Technology-enabled reform in a nontraditional mental health service for eating disorders: Participatory design study | Eating disorders | 2021 |
| Wiberg, Ghaderi, Danielsson, Safarzadeh, Parling, Carlbring, Jansson and Welch | Internet-based cognitive behavior therapy for eating disorders – Development and feasibility evaluation | Eating disorders | 2022 |
| Schmitt and Yarosh | Participatory design of technologies to support recovery from substance use disorders | Substance use disorders | 2018 |
| Derks, Klaassen, Westerhof, Bohlmeijer and Noordzijl | Development of an Ambulatory Biofeedback App to Enhance Emotional Awareness in Patients with Borderline Personality Disorder: Multicycle Usability Testing Study. | Borderline personality disorder | 2019 |
| Flobak, Nordby, Guribye, Kenter, Nordgreen and Lundervold | Designing Videos with and for Adults with ADHD for an Online Intervention: Participatory Design Study and Thematic Analysis of Evaluation | ADHD | 2021 |
| Terlouw, van ’t Veer, Prins, Kuipers and Pierie | Design of a digital comic creator (it's me) to facilitate social skills training for children with autism spectrum disorder: Design research approach | Autism spectrum disorder | 2020 |
| Jonathan, Dopke, Michaels, Bank, Martin, Adhikari, Krakauer, Ryan, McBride, Babington, Frauenhofer, Silver, Capra, Simon, Begale, Mohr and Goulding | A smartphone-based self-management intervention for bipolar disorder (livewell): User-centered development approach | Bipolar disorder | 2021 |
| Gammon, Strand and Eng | Service users' perspectives in the design of an online tool for assisted self-help in mental health: A case study of implications | Various mental health conditions (not specified which) | 2014 |
| Grim, Rosenberg, Svedberg and Schön | Development and usability testing of a web-based decision support for users and health professionals in psychiatric services. | Various mental health conditions (not specified which) | 2017 |
| Vilardaga, Rizo, Zeng, Kientz, Ries, Otis and Hernandez | User-centered design of learn to quit, a smoking cessation smartphone app for people with serious mental illness | Various mental health conditions (major depression and schizophrenia) | 2018 |
| Silva and Bueno | ComPasS: A personal organization mobile app for individuals with mental disorders | Various mental health conditions (not specified which) | 2018 |
| Hackett, Mulvale and Miatello | Co-designing for quality: Creating a user-driven tool to improve quality in youth mental health services. | Various mental health conditions (not specified which) | 2018 |
| Mulvale, Moll, Miatello, Murray-Leung, Rogerson and Sassi | Co-designing Services for Youth With Mental Health Issues: Novel Elicitation Approaches | Various mental health conditions (including depressions, anxiety, eating disorders, and psychotic disorders) | 2019 |
| Maathuis, Niezen, Buitenweg, Bongers and van Nieuwenhuizen | Exploring Human Values in the Design of a Web-Based QoL-Instrument for People with Mental Health Problems: A Value Sensitive Design Approach | Various mental health conditions (major psychiatric problems, people treated in forensic psychiatry, and people who are supported by homelessness services) | 2019 |
| Sanin, Spong and McRae | Creative wellbeing. Prototyping an arts-health practice program for mental health recovery | Various mental health conditions (not specified which) | 2021 |
| Van der Meer, Jonker, Wadman, Wunderink, van Weeghel, Pijnenborg and van Setten | Targeting Personal Recovery of People With Complex Mental Health Needs: The Development of a Psychosocial Intervention Through User-Centered Design | Various mental health conditions (including schizophrenia, schizoaffective disorder, autism, bipolar disorder, borderline personality disorder, anxiety, and depression) | 2021 |
| Bongers, Buitenweg, van Kuijk, van Nieuwenhuizen | I Need to Know: Using the CeHRes Roadmap to Develop a Treatment Feedback Tool for Youngsters with Mental Health Problems | Various mental health conditions (not specified which) | 2022 |
| Bos, von Klipstein, Emerencia, Veermans, Verhage, Snippe, Doornbos, Hadders-Prins, Wichers, Riese | A Web-Based Application for Personalized Ecological Momentary Assessment in Psychiatric Care: User-Centered Development of the PETRA Application | Various mental health conditions (mood, psychotic, or anxiety disorders) | 2022 |
